# Glycoursodeoxycholic acid regulates bile acids level and alters gut microbiota and glycolipid metabolism to attenuate diabetes

**DOI:** 10.1080/19490976.2023.2192155

**Published:** 2023-03-26

**Authors:** Bingting Chen, Yu Bai, Fenglian Tong, Junlin Yan, Rui Zhang, Yewei Zhong, Huiwen Tan, Xiaoli Ma

**Affiliations:** a College of Pharmacy, Xinjiang Medical University, Urumqi, China; b Nanshan Hospital, The First Affiliated Hospital of Guangzhou University of Chinese Medicine (Shenzhen Nanshan Hospital of Chinese Medicine), Shenzhen, China; c The Fourth Affiliated Hospital of Xinjiang Medical University, Urumqi, China

**Keywords:** Type 2 diabetes, bile acids, gut microbiota, glycoursodeoxycholic acid

## Abstract

Accumulating evidence suggests that the bile acid regulates type 2 diabetes mellitus (T2DM) through gut microbiota-host interactions. However, the mechanisms underlying such interactions have been unclear. Here, we found that glycoursodeoxycholic acid (GUDCA) positively regulates gut microbiota by altering bile acid metabolism. GUDCA in mice resulted in higher taurolithocholic acid (TLCA) level and *Bacteroides vulgatus* abundance. Together, these changes resulted in the activation of the adipose G-protein-coupled bile acid receptor, GPBAR1 (TGR5) and upregulated expression of uncoupling protein UCP-1, resulting in elevation of white adipose tissue thermogenesis. The anti-T2DM effects of GUDCA are linked with the regulation of the bile acid and gut microbiota composition. This study suggests that altering bile acid metabolism, modifying the gut microbiota may be of value for the treatment of T2DM.

## Introduction

Type 2 diabetes mellitus (T2DM) is a complex polygenic disease associated with insulin resistance and pancreatic β-cell dysfunction^1^. In spite of the uncertainty surrounding the pathogenesis of T2DM, more and more studies have found that bile acids (BAs) and gut microbiota may be involved. In recent years, the gut microbiota is shown to play an important part in the biotransformation and reabsorption of BAs, and acts on host glycolipid and energy metabolism through co-metabolism of BAs^2^. Sun et al^3^ showed that intestinal flora and bile acid metabolic pathways are the key mechanisms mediating the hypoglycemic effects of metformin. Therefore, the modulation of host lipid metabolism disorders by BAs-gut microbiota co-metabolism has become a new model for early intervention in type 2 diabetes.

Co-metabolic networks between BAs-gut microbiota and the host is considered therapeutic targets for various metabolic diseases. It has been found^4^ that dysbiosis of the intestinal flora leads to a decrease in secondary bile acid production and thus a decrease in activation of bile acid receptors, which further leads to dysregulated glucose metabolism and T2DM disease. Many bacteria, especially some Clostridium perfringens, have been shown to be active in the conversion of primary bile acids to secondary bile acids^5^. Bile acids and microbiota interact in a bidirectional manner. Through their ability to promote the development of bacteria associated with bile acid metabolism as well as curb the growth of bile-sensitive germs, bile acids reshape the microbial community in the intestine. When the flow of bile is blocked, bacteria overgrow and overtake the small intestine in biliary obstruction, and the administration of bile acid can reverse the phenotype^6^. These studies suggest a dynamic interaction between host bile acids and microbial populations in the gut. Therefore, monitoring the composition of bile acids and gut microbiota in human can provide potential help for early diagnosis and treatment of T2DM.

After transplantation of intestinal flora from patients with polycystic ovary syndrome into mice using fecal transplantation technique, it was found that the mice showed some degree of disruption of ovarian function and disturbance in the metabolic conversion of bile acids in vivo, Treatment with glycine deoxycholic acid (GDCA) could alter bile acid metabolism and intestinal flora to improve the disease^7^. Therefore, targeting bile acids to modulate bile acid-gut microbiota could be a potential treatment for metabolic diseases. The efficacy of glycoursodeoxycholic acid (GUDCA) therapy to delay disease progression has been demonstrated^8,9^, and oral supplementation of GUDCA may have potential translational value in the clinical management of T2DM^3^, but whether GUDCA can improve glucolipid metabolism in diabetes through modulation of bile acid-gut microbiota metabolism has not been reported.

In the present study, we investigated the differences of bile acids and intestinal microflora in type 2 diabetic patients with normal controls by metabonomics-high-throughput sequencing. Then, the effects of GUDCA on glucose and lipid metabolism in db/db mice were further studied and the underlying mechanisms were elucidated. Results showed that GUDCA is effective at intervening T2DM by modulating bile acids-gut microbiota, which providing insights into the mechanism of its action as well as guiding future studies about its prospective applications in the therapy of metabolic disorders.

## Results

### Bile acids and gut microbiota alterations between T2DM patients and healthy controls

**Figure 1.**
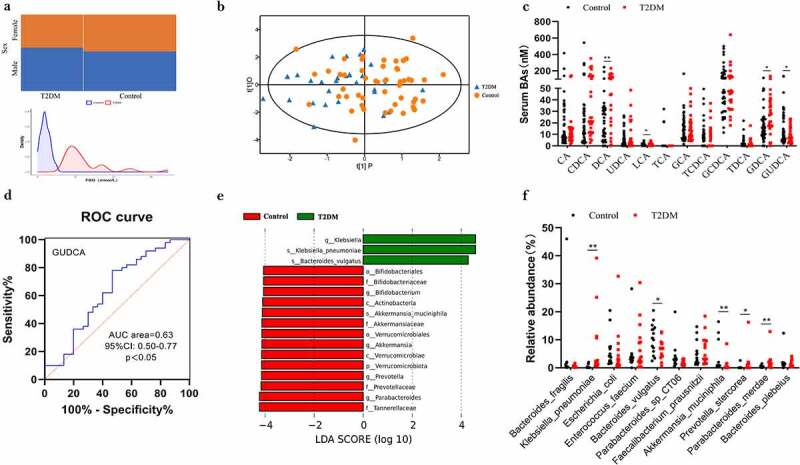
Profile of the bile acids and gut microbiota in individuals with T2DM. **a** Sex proportions and FBG distribution in T2DM and Control. **b** OPLS-DA plot of bile acids levels in T2DM (blue) and Control (orange). **c** Determination of serum bile acids in T2DM and Control group. P values were analyzed by two-tailed Mann–Whitney U-test and data were presented as medians. **d** ROC of GUDCA in predicting T2DM. AUC: area under curve. **e** Analysis with the linear discriminant analysis (LDA). Green indicates enriched taxa in the T2DM group. Red indicates enriched taxa in the Control group. **f** Different species abundance of T2DM and Control based on metagenomics data. *n*=15 individuals/groups. *p＜0.05，**p＜0.01.

To investigate how bile acids and intestinal flora are altered in the organism of T2DM patients, we collected serum from 30 patients and stool from 15 patients. In two independent cohorts (Figure 1(a); Table S1), 29 bile acids were detected. Orthogonal projections to latent structures- discriminant analysis (OPLS-DA) showed that there was a trend to differentiate the bile acid metabolic profile between the T2DM and normal groups (Figure 1(b)). Qualitative and quantitative analysis of bile acid species indicated that deoxycholic acid (DCA), lithocholic acid (LCA) and glycodeoxycholic acid (GDCA) were considerably elevated in the T2DM group compared with the control group in the serum. Notably, we observed that glycoursodeoxycholic acid (GUDCA) was significantly decreased in T2DM group (Figure 1(c)). In addition, we performed a receiver-operating characteristic (ROC) analysis and observed that the proportion of GUDCA had good performance in predicting T2DM subjects (AUC = 0.63, 95%CI:0.50–0.77, p＜0.05) (Figure 1(d)). ACE, Chao1, and Shannon indices revealed no notable differences between the two groups in terms of α diversity (Figure S1(a)). Notably, the β-diversity of microbial communities in the T2DM group was significantly lower compared to the normal group, indicating that the T2DM individual communities were more homogeneous (p < 0.05; Figure S1(b)). Analysis with the linear discriminant analysis (LDA) suggested that *Akkermansia muciniphila* was keystone species in the normal group while *Klebsiella pneumoniae* was considered as the potential biomarker for the T2DM (Figures 1(e)). Detection of abundances at the species level further supported the above observation, in other words, less abundance of *B. vulgates and A. muciniphila* but more of *K. pneumoniae* was showed in T2DM group (Figure 1(f)).

### GUDCA regulates blood glucose and lipid levels

**Figure 2.**
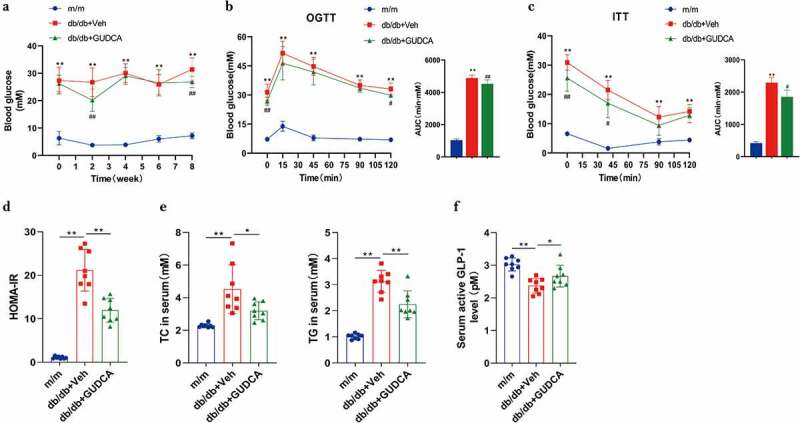
GUDCA supplementation had therapeutic effects in improving glucose tolerance. **a** Fasting blood glucose. **b, c** OGTT, ITT and AUC. Vehicle or GUDCA-treated (100 mg/kg/d) mice on db/db mice for 8 weeks. *n*=10 mice in m/m group, *n*=9 mice in db/db+veh and db/db+gudca groups. All *P* values were analyzed by two-tailed Student’s t-test, **p< 0.01 versus m/m; #p< 0.05, ##p< 0.01 versus db/db+veh. All data are presented as the mean ± sd. **d** HOMA-IR. **E** TC and TG in the serum. **f** Serum active GLP1 levels. *n*=8 mice/group. All *P* values were analyzed by two-tailed Student’s t-test, *p< 0.05, **p< 0.01. All data are presented as the mean ± sd.

To explore the impact of GUDCA on metabolic diseases, db/db mice were gavaged with GUDCA for 8 weeks and their glucose and lipid levels were observed. We found that there was no alteration in food intake, water intake and body weight gain (Figures S2(a-b)). After GUDCA supplementation, db/db+GUDCA mice showed a significant decrease in blood glucose at weeks 2 and 8 (Figure 2(a)). At the same time, glucose tolerance test (GTT) and insulin tolerance test (ITT) results, insulin levels, and HOMA-IR indices in db/db+GUDCA mice were decreased compared with db/db+Veh group (Figures 2(b-d), S2(c)). Research also indicated that lipid levels (TC, TG) in serum had significantly decreased in db/db+GUDCA mice compared with db/db mice. (Figure 2(e)). Furthermore, we found that GUDCA was able to reduce the level of serum GLP-1 compared with db/db+Veh group. (Figure 2(f)).

### Liver lipid profiles, oxidative stress and histopathology

**Figure 3.**
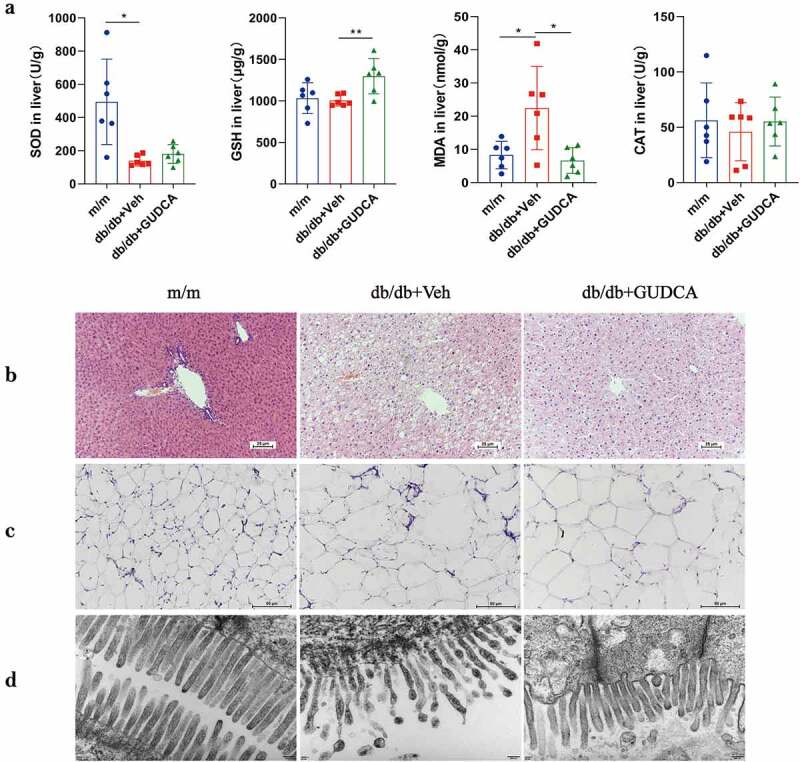
The physiological changes in m/m, db/db+veh and db/db+gudca. **a** Oxidative stress in liver. All *p* values were determined by two-tailed Student’s t-test, *p< 0.05, **p< 0.01. All data are presented as the mean ± sd. *n*=6 mice/group. **b** Representative images of H&E staining of Liver, scale bars, 25μm **c** Representative images of H&E staining of WAT, scale bars, 50μm. **d** Electron microscope of Ileum, Scale bars,50000x.

The levels of serum alanine aminotransferase (ALT) and aspartate aminotransferase (AST) were decreased after GUDCA supplementation, indicating that liver function impairment was improved in db/db mice to some extent (Figure S2(d)). TC and TG levels in the livers of mice fed with GUDCA were decreased significantly compared with the db/db+Veh group (Figure S2(e)). Diabetes, for instance, is a result of oxidative stress, which plays an integral role in the development of a wide range of diseases. We found that the SOD levels were significantly decreased and MDA levels were significantly increased in db/db mice compared with m/m mice, indicating that a certain degree of oxidative damage existed in the liver tissues of db/db mice. At the same time, GUDCA administration was able to improve the oxidative stress damage to some extend by elevating GSH and decreasing MDA levels (Figure 3(a)). However, there is no significantly increased in SOD and CAT in liver after GUDCA administration. GUDCA-treated mice displayed improved morphology in their livers. A histological examination of liver specimens found cellular swelling and lipid vacuoles in the db/db group as compared to the m/m group. Hepatocytes in the GUDCA group were more normal in structure, with intact cell morphology and significantly fewer vacuoles, tending to be normal hepatocytes (Figure 3(b)). The above observations indicate that GUDCA could reduce oxidative stress injury to a certain extent and have a protective effect on liver tissue.

### Dynamic change of BAs profile in serum

**Figure 4.**
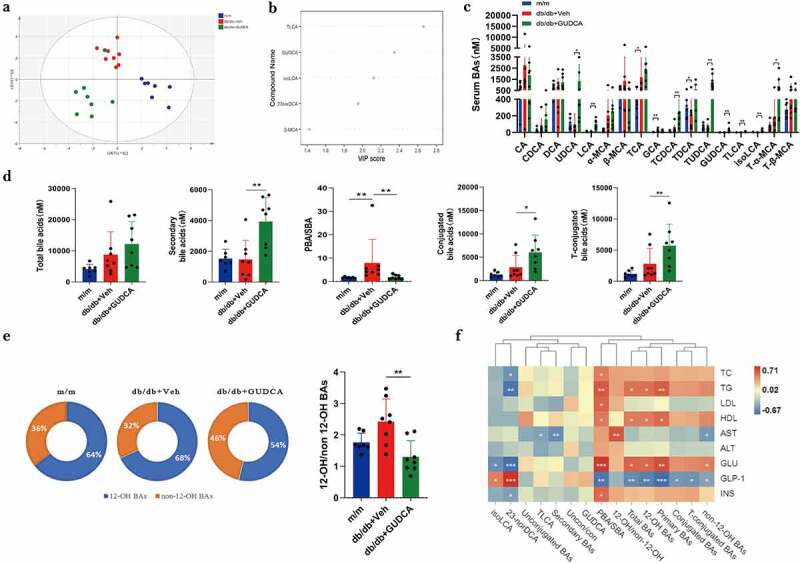
Dysregulated BA profiles in m/m, db/db+veh and db/db+gudca. **a** Orthogonal partial least squared-discriminant analysis (OPLS-DA) scores plot of serum BA profiles showing the group of m/m (blue) group, db/db+veh (red) and db/db+gudca (green). **b** the variable importance in projection (VIP) scores from OPLS-DA model based on the serum BA profiles between the db/db+veh and db/db+gudca group. **c, d, e** Profiles of BAs in the db/db+veh group. *p< 0.05, **p< 0.01. All data are presented as the mean ± sd. **f** Heatmap of spearman correlation association between serum BAs and blood indicators from three groups. *p< 0.05.

Ultra-performance liquid chromatography tandem mass spectrometry (UPLC-MS/MS) was utilized to assess serum BAs profiles in the mice. As can be seen from the results of the OPLS-DA model, a clear separation between the m/m mice and db/db+Veh mice were observed. Similarly, a separation trend between the db/db+Veh group and db/db+GUDCA group was also discovered (Figure 4(a)). As a result of the serum BA profiles of the OPLS-DA, there was a significant difference between groups db/db+Veh and db/db+GUDCA because TLCA ranked higher in the VIP scores (Figure 4(b)). A summary of serum BA concentrations is shown in Table S2. Results showed that TCA、GCA、TCDCA、TDCA were significantly increased in db/db+Veh group. Compared with the m/m group, the ratios of PBAs to SBAs (PBA/SBA ratio) were elevated in db/db mice. Further, the levels of secondary BAs, conjugated BAs and taurine BAs increased, whereas PBA/SBA ratio decreased after GUDCA treatment (Figure 4(c-d)).In addition, we found that UDCA、LCA、TCDCA、TUDCA、GUDCA、TLCA, isoLCA, *T*-α-MCA were significantly increased in the db/db+GUDCA group and furthermore, all these differential BAs belonged to the category of non-12α-OH BAs (Figure 4(e)). 10 of the 29 bile acids detected in db/db+Veh group were upregulated and 19 were downregulated, and an increase in 18 bile acids could be observed after GUDCA administration, with significant differences in metabolite levels among three groups (Figure S3). Then, Spearman correlation analysis were executed to illuminate the coefficients between serum BAs and serum indicators for the three groups. In general, a majority of the BAs were significantly negatively correlated with GLP-1. Moreover, PBA/SBA had a positive correlation with blood biochemical parameters, including TC、TG、LDL、HDL、GLU、INS (Figure 4(f)).

### Changes in intestinal mucosal barrier

The levels of diamine oxidase (DAO) and D-lactic acid (D-LA) in the serum of mice in db/db+Veh group were increased significantly (*p* < 0.01) compared with the m/m group. The DAO and D-LA levels in the db/db+GUDCA group were 20.8% and 24.3% lower than in the db/db+Veh group (*p* < 0.01), respectively (Figure S4(a)). Transmission electron microscopy was applied to inspect the function of ileum in mice. The results revealed that the microvilli in the m/m group were intact and tightly arranged, whereas the microvilli were disorganized and incomplete in the db/db group, with coupled microvilli that were dislodged into the intestinal lumen. Surprisingly, the mice treated with GUDCA had lower injury in ileum, which indicated GUDCA could improve the abnormal intestinal mucosal barrier and protect the imbalance of intestinal homeostasis (Figure 3(d)).

### The structure of the gut microbiota

**Figure 5.**
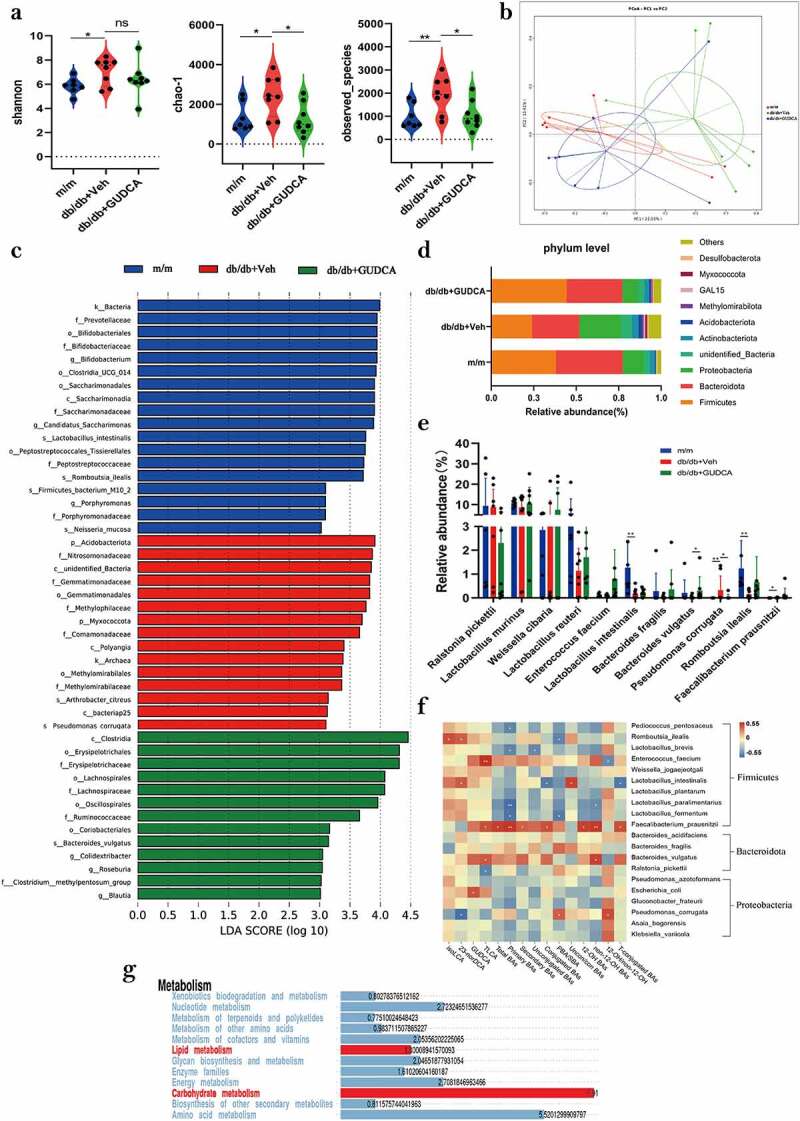
GUDCA modulates the composition of gut commensal bacteria. **a** α-diversity of the gut microbiota, as indicated by the Shannon, Chao1 indices and observed species. **b** Principle coordinate analysis (PCOA) plot generated using OTU metrics based on the Binary-Jaccard similarity for m/m, db/db+veh and db/db+gudca groups. **c** Taxonomic cladogram generated from LEfSe of metagenomic sequencing data. Blue indicates enriched taxa in the m/m group. Red indicates enriched taxa in the db/db+veh group. Green indicates enriched taxa in the db/db+gudca group. **d** the relative abundance of phylum level in the db/db+veh group. **e** Dysregulated gut microbiota in the db/db+veh group. *p< 0.05, **p< 0.01. P values were determined by two-tailed Mann–Whitney U-test and data are presented as the mean ± sd. **f** Heatmap of spearman correlation coefficients between serum BAs and blood biochemical parameters from all samples in the three groups. *p< 0.05(spearman’s correlation with the post hoc correction using the Holm method). **g** Kyoto Encyclopedia of Genes and Genomes annotation of key altered metabolic pathways in three groups.

The Venn diagram showed that 7971 OTUs were shared across the three groups (Figure S4(b)). There was significant difference between db/db+Veh and db/db+GUDCA group in α diversity, as indicated by the decrease of chao-1 indices and observed_species (Figure 5(a)). The β-diversity indicated that the composition and abundance of microbiota after GUDCA administration tended to the m/m mice, which was significantly different from the db/db+Veh group (*p* < 0.05; Figure 5(b)). Linear discriminant analysis (LDA) displayed that the db/db+Veh group was characterized by *Pseudomonas corrugata* and *Arthrobacter citreus*, then *Bacteroides vulgatus* was considered as the key species in the db/db+GUDCA group (Figure 5(c)). Meanwhile, in the analysis of species abundance in each group, we found that the abundance of *Pseudomonas corrugata* decreased and *Bacteroides vulgatus* increased in db/db+GUDCA group compared with the model group (Figure 5(e)). In addition, we analyzed the levels of short-chain fatty acids in the feces by using gas chromatography. The results revealed that the levels of acetic acid and propionic acid decreased in the db/db+Veh group, and GUDCA administration was able to reverse the trend of decreasing acetic acid and propionic acid (Figure S4(c)). The *Firmicutes* and *Bacteroidota* are the dominant phylum in the intestine that produce SCFAs, and in this study, the relative abundance of the *Firmicutes* and *Bacteroidota* was found to be elevated in the GUDCA group compared to the model group (Figure 5(d)).

Spearman correlation analysis of intestinal flora and bile acids (Figure 5(f)) showed that *Faecalibacterium_prausnitzii* correlated with multiple bile acids. *Bacteroides vulgatus* had positively correlated with the levels of TLCA, non 12α-OH BAs, and *Pseudomonas corrugata* had a positive correlation with elevated relative abundance of 12α-OH/non 12α-OH BAs, PBA/SBA in db/db+Veh group mice. KEGG analysis revealed that lipid metabolism and carbohydrate metabolism were the key metabolic pathways influenced by the gut microbiota changes found in the mice.

### GUDCA ameliorated metabolism by promoting fat thermogenesis

**Figure 6.**
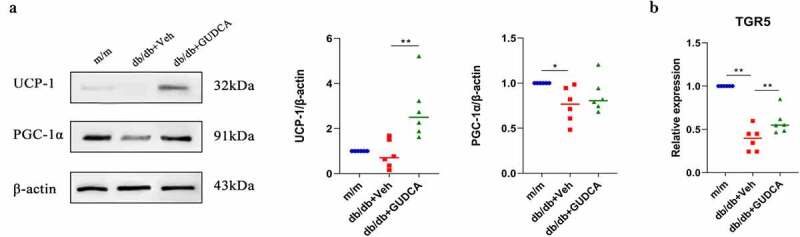
GUDCA ameliorated metabolism by promoting fat thermogenesis. A: Western blot analysis of UCP1 and PGC-1 in white adipose tissues. B: Relative mRNA expression of TGR5. *p< 0.05, **p< 0.01. All data are presented as the mean ± sd.

In order to investigate whether GUDCA can improve obesity in db/db mice by affecting adipose function, white adipose from mice was taken for study. The white adipocytes in the db/db group were swollen and ruptured with increased lipid droplets, surprisingly, the cell morphology was intact and the adipocytes were not ruptured after GUDCA administration (Figure 3(c)). UCP1 was significantly higher in the db/db+GUDCA group compared with the db/db+Veh group (*p* < 0.01). Also, we found that PGC-1α in the white adipose tissue of db/db+Veh mice was decreased compared with the m/m group (*p* < 0.05), and GUDCA had a tendency to elevate PGC-1α but no significant difference (*p* > 0.05; Figure 6(a)). In addition, we also found elevated expression of white adipose TGR5 on mRNA (Figure 6(b)).

## Discussion

The close association between bile acid metabolism and gut microbiota plays an important role in the regulation of immune function, maintenance of host nutrient metabolism and energy balance, and health of the organism^10^. Currently, the research of gut microbiota and bile acid metabolism for the treatment and prevention of metabolic diseases, including obesity, diabetes, inflammation and NAFLD^11,12^, has become a research direction. In this study, we investigated the differences between bile acids and gut microbiota in type 2 diabetes mellitus and normal subjects by means of metabolomics-high-throughput sequencing, and then further investigated the effects of GUDCA on glucolipid metabolism in db/db mice and elucidated the potential mechanisms.

It has been demonstrated that the characteristics of BA show significant changes in patients with type 2 diabetes^13,14^. In present study, we used multivariate statistical analysis of serum bile acids in type 2 diabetes and found a significant decrease in GUDCA in the serum of diabetic patients compared to normal subjects, which can be used as a potential marker for clinical diagnosis and treatment. We also found that there was a dynamic interaction between bile acids and microbial populations in the intestine. Using metformin to treat patients with T2DM, Sun et al^3^found that levels of GUDCA increased in the gut, which was in line with our research. They also indicated that metformin was able to reduce *Bacteroides fragilis* in the gut, however, we found that a corresponding decrease in *Bacteroides vulgatus* occurred in the intestine of type 2 diabetic patients. The use of bile acids as targeted agents for the treatment of hepatobiliary diseases has become a hot research topic in recent years after UDCA was approved by the U.S. Food and Drug Administration (FDA) for the treatment of primary cholangitis^15^. Feeding cholic acids (CA) to rats was able to significantly alter the microbiota at the phylum level, leading to an increase in *Firmicutes* and a corresponding decrease in *Bacteroidota* ^16^. A short-term study of UDCA revealed that it was able to increase hepatic triglyceride (TG) levels^17^ and tauroursodeoxycholic acid (TUDCA) has been shown to be effective in preventing inflammation and improving insulin sensitivity^18,19^.

Supplementation with glycoursodeoxycholic acid (GUDCA) can come to inhibit the intestinal FXR axis, which can reduce blood ceramide levels and thus reduce atherosclerosis in ApoE-/- model mice on a high cholesterol diet^20^. In this study, GUDCA treatment was found to be therapeutically effective in the metabolic disorders of db/db mice. GUDCA decreased blood glucose and reduced serum alanine aminotransferase (ALT) and aspartate aminotransferase (AST) levels in db/db mice, indicating that GUDCA has a hepatoprotective effect. GUDCA was associated with glucose control^21^, which was also indicated in our study. We also found that GUDCA significantly reduced the serum and liver levels of TC and TG in db/db mice after continuous administration. Insulin resistance is a key pathogenic factor in the development of diabetes mellitus, so, improving insulin resistance plays an important role in the treatment of diabetes mellitus^22^. The findings indicated that serum insulin levels were elevated in db/db mice, GUDCA decreased serum insulin levels and HOMA-IR in db/db mice. High levels of insulin concentrations could be attributed to increased insulin secretion or decreased insulin clearance^23^, oxidative stress is considered to be one of the most critical mechanisms of insulin resistance^24^. Elevated levels of oxidative stress may be a major deleterious factor contributing to insulin resistance, β-cell dysfunction, impaired glucose tolerance, and dyslipidemia^25–27^. In this study, SOD activity decreased and MDA content increased in the liver of db/db mice compared with the normal group was found, and then GUDCA could increase GSH and decrease MDA content, effectively improving oxidative stress damage and reducing insulin resistance in the organism.

Changes in the composition of the BA pool are associated with metabolic disorders^28^. In our experiments, serum bile acids in db/db mice underwent significant changes, mainly in the upregulation of UDCA with its taurine and glycine conjugates (TUDCA, GUDCA) and LCA with its conjugates (isoLCA, TLCA) after eight weeks of GUDCA intervention. TLCA, an agonist of TGR5, made the largest contribution in the db/db+Veh group and db/db+GUDCA group. TGR5 is a receptor that positively regulates energy metabolism, and studies have shown that secondary bile acids have a higher affinity for TGR5 than primary bile acids. By activating TGR5 are able to induce GLP-1 secretion in the intestine and thermogenic energy expenditure in brown fat and skeletal muscle, thereby improving glucose and energy homeostasis^29^. Thus, we hypothesized that after administration of GUDCA, GUDCA is changed to UDCA in vivo by choline hydrolase (BSH), UDCA is rapidly metabolized to LCA by bacterial hydroxysteroid dehydrogenase. Exogenous supplementation of GUDCA to regulate glucolipid metabolism may be associated with increased TLCA.

The gut microbiota plays an irreplaceable role in human physiology and pathology^30^. Microbial dysbiosis includes imbalance in the distribution of bacterial populations and impaired bacterial metabolic activity, which can increase intestinal permeability and thus lead to multiple disorders^31,32^. In a correlation analysis between bile acids and gut microbiota, the abundance of *B. vulgatus* was found to be positively correlated with TLCA. BSH is present in *B. vulgatus*, and BSH catalyzes the hydrolysis of conjugated bile salts to form amino acids and free bile acids, thus serving to maintain the balance of bile acid metabolism^33^. White fat is a type of adipose tissue in the human body, and the accumulation of large amounts of white fat leads to obesity. Virtue^34^ showed that indole-3-carboxylic acid (I3CA) and indole, tryptophan metabolites of gut microbiota, can significantly inhibit miR-181 expression thereby regulating white fat for weight loss. A study by Wu found that intestinal HIF-2α-specific knockdown could promote white fat thermogenesis and improve obesity by modulating the intestinal lactate-*B. vulgatus*-bile acid-adipose TGR5 signaling pathway^35^. Several researches have suggested that GUDCA can ameliorates diseases by regulating FXR^3,20,36^. Nevertheless, in present research, we found that GUDCA could activate TGR5 expression on mRNA in white adipose tissue and promote white adipose thermogenesis. Further, we hypothesized that GUDCA may provide a new target and intervention strategy for the prevention and treatment of obesity and related metabolic diseases by regulating the TLCA-*B.vulgatus*-adipose TGR5 signaling pathway to promote adipose thermogenesis and improve glucolipid metabolism. However, we still need to do a lot of experiments to test our hypothesis.

There are several limitations in our study. First, all subjects in the study were recruited in a single region. Different dietary can have an impact on the composition of the gut microbiota. Moreover, the number of feces samples was small, which make the data are not representative enough. Second, it remains unclear whether altered GUDCA level directly mediates changes in gut microbiota on diabetes prevention. To understand how gut microbiota protects against diabetes as a result of GUDCA-related bacteria, future studies in germ-free mouse fecal microbiota transplantation need to be conducted. Third, there is a disparity in gut microbiota composition between mice and humans^37,38^, which makes it difficult to clarify how GUDCA influences gut microbiota in humans. In addition, GUDCA’s safety and effectiveness, however, remain to be determined in the future due to the lack of clinical evidence. In conclusion, GUDCA supplementation modulates the abundance of gut microbiota, upregulate beneficial bacteria, and alter the bile acid metabolic profile to some extent. Additionally, GUDCA can also activate white fat TGR5 and increase lipid thermogenesis. GUDCA signaling is expected to be a prospective target for the therapy of human metabolic diseases.

## Materials and methods

### Human subjects

Human serum samples were collected from 30 people with T2DM and 50 healthy subjects. For serum extraction, all blood samples were centrifuged for 20 minutes at 3500 rpm after 30 minutes at room temperature.15 individuals with T2DM and 15 controls were recruited to collect feces. Feces samples were collected with a sterile spoon and stored at − 80°C until analysis. All of the subjects enrolled satisfied the diagnostic criteria by American Diabetes Association (ADA) in 2021: FPG≥7.0 mmol/L or 2-h PG ≥11.1 mmol/L or A1C ≥ 6.5%. The exclusion criteria were: type 1 diabetes; gestational diabetes; pregnancy; no antibiotic and probiotics use within 3 months; gastrointestinal diseases; mental illness; and alcoholism. Clinical parameters were determined at The Fourth Affiliated Hospital of Xinjiang Medical University. The demographic characteristics, lipid analysis and insulin relative indicators of cohort involved in the research are listed in Supplementary Table 1. The study protocol was approved by the Ethics Committees of Xinjiang Medical University (Permission number:20140304–133). All participants provided written informed consent.

### Mice

Male C57BL/Ksj-db/db mice (35–45 g, 6–8 weeks old) and C57BL/Ksj-m/m mice (20-23 g, 6–8 weeks old) were purchased from Changzhou Cavens Model Animal Co.,Ltd with the permission number SCXK 2016–0010. After two weeks of adaptive feeding under a 12 h light/dark cycle at 21 ± 2°C with enough food and water, the mice were randomly divided into 3 groups. The study protocol followed international ethical guidelines and was approved by the Animal Care and Use Committee of Xinjiang Medical University.

The m/m mice (*n* = 10) and db/db mice (*n* = 10) were fed a standard chow diet and drinking water with vehicle for 8 weeks. At the same time, ten db/db mice were fed a standard chow diet and given 100 mg/kg/d GUDCA (Sigma-Aldrich, Cat# 06863) by gavage for 8 weeks. Weekly measurements of body weight, food and drink intake, and blood glucose were taken during experiments. Stool samples were collected during the last week and stored at−80°C until the analysis. All mice were fasted and water was available for 16 hours before death. The blood samples were collected and centrifuged at 3500rpm for 15 min at 4°C to obtain the serum. Liver, ileum, and white adipose were immediately collected after excision. The tissues were stored at−80°C until further analysis or in 4% paraformaldehyde and 2.5% glutaraldehyde for histological and transmission electron microscopy studies.

### Bile acid analysis

Bile acids were quantified with a UPLC/MS-MS system (Agilent, Thermo Fisher Scientific, USA) with an ESI source ^39^. Taurochenodeoxycholic acid (TCDCA-d4) was used as internal standards. An Acquity BEH C18 column (100 mm × 2.1 mm i.d., 1.7 μm, Waters Corp.) was used at the temperature of 45°C, and the flow rate of 0.4 ml/min for liquid chromatography separation. The solvent of the mobile phase was a mixture of 0.1% acetic acid in water and acetonitrile. The gradient elution was applied and MS detection proceeded in negative mode. A Q Exactive Focus mass spectrometer (Thermo Fisher Scientific) was applied for assay development in Parallel Reaction Monitoring (PRM) mode^40^.

### Multivariate analysis

Orthogonal projections to latent structures-discriminant analysis (OPLS-DA) was used to determine taxonomic changes, and VIP (variable importance) scores were adopted to rank the ability of different taxa to discriminate between different groups^41^. The results of the differential metabolite screening were visualized as a volcano plot. Then, the Euclidean distance matrix was calculated, and the differential metabolites were clustered by the complete chain method and presented as a heat map. Receiver operating characteristic (ROC) curve were plotted and calculated its area under the curve to obtain potential biomarkers (performed using the vegan package in R 3.4.0).

### DNA extraction and preparation

Genomic DNA from human and mouse stool samples was extracted using Magnetic Soil and Stool DNA Kit (Tiangen biotech Co. Ltd., Beijing, China). Degradation and contamination of DNA were monitored on 1% agarose gels. DNA concentrations were measured using a NanoDrop system (Thermo Fisher Scientific), and the DNA molecular size was estimated by agarose gel electrophoresis.

### Metagenomics sequencing

Metagenomics sequencing was measured as previously described^42^. In brief, the V3-V4 region of the bacterial rRNA gene was amplified by polymerase chain reaction (PCR; 98°C for 1 min, followed by 30 cycles of 98°C for 10 s, 50°C for 30 s and 72°C for 30 s and a final extension at 72°C for 5 min) using the primers 515F (5’-GTGCCAGCMCCGCGGTAA-3’) and 806 R(5’-GGACTACHVGGGTWTCTAAT-3’). Mixture PCR products was purified with GeneJET Gel Extraction Kit (Thermo Scientific). Sequences with a primary band size between 400-450bp was selected and then cut the gum to recover the target bands. Sequencing libraries were constructed using Illumina TruSeq DNA PCR-Free Library Preparation Kit (Illumina, USA). The library quality was tested on the Qubit@ 2.0 Fluorometer (Thermo Scientific) and Agilent Bioanalyzer 2100 system. At last, the library was sequenced on an Illumina NovaSeq platform and 250 bp paired-end reads were generated.

### Metagenomics analysis

Paired-end reads from the original DNA fragments are merged by using FLASH (https://www.flash.cn/). Paired-end reads was assigned to each sample according to the unique barcodes. Sequences were analyzed using QIIME (http://qiime.org/) software package, and in-house Perl scripts were used to analyze alpha- (within samples) and beta- (among samples) diversity. Sequences with≥97% similarity were assigned to the same OTUs. We pick a representative sequence for each OTU and use the RDP classifier to annotate taxonomic information for each representative sequence. Alpha diversity analyses (ACE and Shannon) were calculated using Mothurb.1.30.1 (http://.mothur.org/). The relative abundance was evaluated using the vegan package of R software. A heatmap based on the relative OTU abundance was generated using the gplot package of R software and the color of the heatmap is displayed as logarithmic values. Analyses of principal coordinate analysis (PCoA) by Bray-Curtis dissimilarity was performed using Mothurb.1.30.1 (http://.mothur.org/)^43–45^. KEGG pathway was performed using in KEGG Pathway Database (http://www.kegg.jp/kegg/pathway.html)^46^.Spearman correlation analysis was performed, and only correlations with *p* < 0.05 and *r* > 0.5 are displayed.

### Metabolic assays

Glucose tolerance tests were performed after 16–18 h fasting. Blood glucose concentrations were measured with a glucometer, and blood samples were taken from the tail tip at 0, 15, 45, 90 and 120 min after oral glucose (2 g/kg body weight). For the insulin tolerance test, insulin (0.75 U/kg body weight) was administered via intraperitoneal injection after 4 h fasting and tail sampling was performed at 0, 40, 90 and 120 min. All of the ITT and GTT tests were performed at indicated times.

### Biochemical analyses

The total triglyceride (TG), total cholesterol (TC), high-density lipoprotein cholesterol (HDLC), low-density lipoprotein cholesterol (LDLC), alanine transaminase (ALT) and aspartate transaminase (AST) levels in the plasma and the hepatic TC and TG levels were measured with commercially available kits (Jiancheng Institute of Biotechnology, Nanjing, China). The levels of Diamine oxidase (DAO) and D-lactic acid (D-LA) in the serum were assayed using commercially available kits (Sino Best Biological Technology Co., Ltd., Shanghai, China). Insulin (mlbio Co, Ltd., Shanghai, China) was determined by enzyme-linked immunosorbent assay kits for mice. The levels of superoxide Dismutase (SOD), glutathione (GSH), malondialdehyde (MDA) and catalase (CAT) in the liver were assayed to evaluate the oxidative stress using commercially available kits (Solarbio Co, Ltd., Beijing, China).

### Serum active GLP-1 detection

Vehicle- or GUDCA- (100 mg/kg/d) treated mice for 3 weeks received sitagliptin (25 mg/kg, DPP4 inhibitor) by gavage 1 h before serum collection and oral glucose (2 g/kg) challenge 15 min before serum collection^3^. Serum active GLP1 levels were measured by GLP-1 ELISA assay kit (Shanghai Enzyme-linked Biotechnology Co., Ltd).

### Histological analysis

Liver and adipose tissues were fixed in 4% paraformaldehyde, and paraffin sections were cut, 3 mm thick were stained with hematoxylin-eosin (HE) and Sirius red. The stained samples were observed with an optical microscope at 200× magnification. Ileum was fixed in 2.5% glutaraldehyde for transmission electron microscopy.

### Fecal SCFA quantification

Vortex 1 mL of acetic acid, propionic acid and butyric acid, dilute them with ultrapure water, and add the appropriate amount to a centrifuge tube containing 2-ethylbutyric acid and mix. 600 μL ethyl acetate and 120 μL IS (500 μg/mL 2-ethylbutyric acid) was added to 20 mg of feces, and the sample was homogenized for 10 min, and then centrifuged at 12000rpm at 4°C for 10 min. An appropriate amount of fecal supernatant was transferred to a 1.5 mL EP tube and passed through a 0.22 μm filter membrane, which was subsequently used to top up the sample. The fecal SCFA analysis was performed by gas chromatography (GC) analysis (Shimadzu, 2010plus). Chromatographic separation was achieved on RTS-WAX column (30 m × 250 μm × 0.25 μm; GL science) coupled to a flame ionization detector (FID). The initial temperature was 100°C, and the temperature was increased to 250°C at 20°C/min and maintained for 1 min. The FID temperature and inlet temperature were set at 230°C and 250°C, respectively. An autosampler (AOC-20i) was employed with an injection volume of 10 μL.

### Real-time PCR analysis

Real-time qPCR analysis was performed using the SYBR Green PCR master mix (Invitrogen) using the ABI 7500 real-time PCR system (Applied Biosystems). A standard phenol-chloroform extraction was performed to isolate total RNA from frozen tissues with Trizol reagent. cDNA was synthesized from 2 μg of total RNA with a PrimeScript^TM^ RT reagent Kit (Takara bio, Beijing, China)^47^. The sequences of the forward and reverse primer for β-actin were 5’-GGCTGTATTCCCCTCCATCG-3’ and 5’-CCAGTTGGTAACAATGCCATGT-3’. The sequences of the forward and reverse primer for TGR5 were 5’-TGCTTCCTAAGCCTACTACT-3’ and 5’-CTGATGGTTCCGGCTCCATAG −3’ respectively. The amplification thermal cycling conditions were as follows: 95°C for 30 s, 40 cycles at 95°C for 5 s and 60°C for 34 s.

### Western blot analysis

UPC1 (1:5,000, Ab209483) and PGC-1α (1:1,000, Ab188102) were purchased from Abcam Co. Ltd., CA, USA. The primary antibody against b-actin (1:10000, bs-0061 R) and secondary antibodies against rabbit (1:50000, bs-0295 G-HRP) were purchased from Bioss Co. Ltd., Beijing, China. Adipose tissues were homogenized in RIPA buffer with protease and phosphatase inhibitors; the protein extracts were separated by SDSPAGE electrophoresis and transferred to a PVDF membrane. Membranes were blocked with 5% nonfat milk for 2 h at room temperature in TBST buffer (10 mM Tris, 150 mM NaCl, pH 7.6, and 0.1% Tween 20) and probed with primary antibodies overnight at 4°C. Membranes were then incubated with horseradish peroxidase-conjugated secondary antibodies. The protein bands were developed using an ECL kit (Biosharp Co. Ltd., Anhui, China). The densitometry analysis of the bands was performed using a gel documentation system (Gel Analyzer, ShineTech, Beijing, China).

### Statistical analysis

Statistical analyses were performed using SPSS 22.0, and results are presented as means ± standard errors or means ± standard deviation. Two-tailed unpaired Student’s t-test and one-way ANOVA with Tukey’s correction were used for all comparisons of mice-related experiments, and a Wilcoxon matched-pairs signed rank test was used for clinical indicators in individuals with T2DM. *P* values<0.05 were considered significant. Correlation analysis of bile acid and gut microbiota were investigated using nonparametric Spearman’s test.

## Supplementary Material

Supplemental Material

## Data Availability

The data that support the findings of this study are available in https://www.ncbi.nlm.nih.gov/bioproject/PRJNA855315, reference number PRJNA855315, and within the article and its supplementary materials.

## References

[1] Mojsak P, Miniewska K, Godlewski A, Adamska-Patruno E, Samczuk P, Rey-Stolle F, Bauer W, Barbas C, Kretowski A, Ciborowski M. A preliminary study showing the impact of genetic and dietary factors on GC-MS-based Plasma metabolome of patients with and without PROX1-genetic predisposition to T2DM up to 5 years prior to prediabetes appearance. Curr Issues Mol Biol. 2021;43(2):513–15. doi:10.3390/cimb43020039. 34209638 PMC8929026

[2] Erawijantari PP, Mizutani S, Shiroma H, Shiba S, Nakajima T, Sakamoto T, Saito Y, Fukuda S, Yachida S, Yamada T. Influence of gastrectomy for gastric cancer treatment on faecal microbiome and metabolome profiles. Gut. 2020;69(8):1404–1415. doi:10.1136/gutjnl-2019-319188. 31953253 PMC7398469

[3] Sun L, Xie C, Wang G, Wu Y, Wu Q, Wang X, Liu J, Deng Y, Xia J, Chen B, et al. Gut microbiota and intestinal FXR mediate the clinical benefits of metformin. Nat Med. 2018;24(12):1919–1929. doi:10.1038/s41591-018-0222-4. 30397356 PMC6479226

[4] Ma Q, Li Y, Li P, Wang M, Wang J, Tang Z, Wang T, Luo L, Wang C, Wang T, et al. Research progress in the relationship between type 2 diabetes mellitus and intestinal flora. Biomed Pharmacother. 2019;117:109138. doi:10.1016/j.biopha.2019.109138. 31247468

[5] Ridlon JM, Kang DJ, Hylemon PB. Bile salt biotransformations by human intestinal bacteria. J Lipid Res. 2006;47(2):241–259. doi:10.1194/jlr.R500013-JLR200. 16299351

[6] Wahlström A, Sayin SI, Marschall HU, Bäckhed F. Intestinal Crosstalk between Bile acids and microbiota and its impact on host metabolism. Cell Metab. 2016;24(1):41–50. doi:10.1016/j.cmet.2016.05.005. 27320064

[7] Qi X, Yun C, Sun L, Xia J, Wu Q, Wang Y, Wang L, Zhang Y, Liang X, Wang L, et al. Gut microbiota–bile acid–interleukin-22 axis orchestrates polycystic ovary syndrome. Nat Med. 2019;25(8):1225–1233. doi:10.1038/s41591-019-0509-0. 31332392 PMC7376369

[8] Vaz AR, Cunha C, Gomes C, Schmucki N, Barbosa M, Brites D. Glycoursodeoxycholic acid reduces matrix metalloproteinase-9 and caspase-9 activation in a cellular model of superoxide dismutase-1 neurodegeneration. Mol Neurobiol. 2015;51(3):864–877. doi:10.1007/s12035-014-8731-8. 24848512

[9] Silva SL, Vaz AR, Diógenes MJ, van Rooijen N, Sebastião AM, Fernandes A, Silva RF, Brites D. Neuritic growth impairment and cell death by unconjugated bilirubin is mediated by NO and glutamate, modulated by microglia, and prevented by glycoursodeoxycholic acid and interleukin-10. Neuropharmacology. 2012;62(7):2398–2408. doi:10.1016/j.neuropharm.2012.02.002. 22361233

[10] Duparc T, Plovier H, Marrachelli VG, Van Hul M, Essaghir A, Ståhlman M, Matamoros S, Geurts L, Pardo-Tendero MM, Druart C, et al. Hepatocyte MyD88 affects bile acids, gut microbiota and metabolome contributing to regulate glucose and lipid metabolism. Gut. 2017;66(4):620–632. doi:10.1136/gutjnl-2015-310904. 27196572 PMC5529962

[11] Song X, Sun X, Oh SF, Wu M, Zhang Y, Zheng W, Geva-Zatorsky N, Jupp R, Mathis D, Benoist C, et al. Microbial bile acid metabolites modulate gut RORγ+ regulatory T cell homeostasis. Nature. 2020;577(7790):410–415. doi:10.1038/s41586-019-1865-0. 31875848 PMC7274525

[12] Jiang T, Xu C, Liu H, Liu M, Wang M, Jiang J, Zhang G, Yang C, Huang J, Lou Z. Linderae radix Ethanol extract alleviates diet-induced Hyperlipidemia by regulating bile acid metabolism through gut microbiota. Front Pharmacol. 2021;12:627920. doi:10.3389/fphar.2021.627920. 33679408 PMC7925880

[13] Sun Y, Zhu M, Zhao H, Ni X, Chang R, Su J, Huang H, Cui S, Wang X, Yuan J, et al. Serum fibroblast growth factor 19 and total Bile acid concentrations are potential biomarkers of Hepatocellular carcinoma in patients with type 2 Diabetes mellitus. Biomed Res Int. 2020;2020:1751989. doi:10.1155/2020/1751989. 32104677 PMC7036095

[14] Shapiro H, Kolodziejczyk AA, Halstuch D, Elinav E. Bile acids in glucose metabolism in health and disease. J Exp Med. 2018;215(2):383–396. doi:10.1084/jem.20171965. 29339445 PMC5789421

[15] Marschall HU, Wagner M, Zollner G, Fickert P, Diczfalusy U, Gumhold J, Silbert D, Fuchsbichler A, Benthin L, Grundström R, et al. Complementary stimulation of hepatobiliary transport and detoxification systems by rifampicin and ursodeoxycholic acid in humans. Gastroenterology. 2005;129(2):476–485. doi:10.1016/j.gastro.2005.05.009. 16083704

[16] Islam KB, Fukiya S, Hagio M, Fujii N, Ishizuka S, Ooka T, Ogura Y, Hayashi T, Yokota A. Bile acid is a host factor that regulates the composition of the cecal microbiota in rats. Gastroenterology. 2011;141(5):1773–1781. doi:10.1053/j.gastro.2011.07.046. 21839040

[17] Mueller M, Thorell A, Claudel T, Jha P, Koefeler H, Lackner C, Hoesel B, Fauler G, Stojakovic T, Einarsson C, et al. Ursodeoxycholic acid exerts farnesoid X receptor-antagonistic effects on bile acid and lipid metabolism in morbid obesity. J Hepatol. 2015;62(6):1398–1404. doi:10.1016/j.jhep.2014.12.034. 25617503 PMC4451470

[18] Kusaczuk M. Tauroursodeoxycholate-bile acid with chaperoning activity: molecular and cellular effects and therapeutic perspectives. Cells. 2019;8(12):1471. doi:10.3390/cells8121471. 31757001 PMC6952947

[19] Zangerolamo L, Vettorazzi JF, Solon C, Bronczek GA, Engel DF, Kurauti MA, Soares GM, Rodrigues KS, Velloso LA, Boschero AC, et al. The bile acid TUDCA improves glucose metabolism in streptozotocin-induced Alzheimer’s disease mice model. Mol Cell Endocrinol. 2021;521:111116. doi:10.1016/j.mce.2020.111116. 33321116

[20] Huang K, Liu C, Peng M, Su Q, Liu R, Guo Z, Chen S, Li Z, Chang G. Glycoursodeoxycholic acid Ameliorates Atherosclerosis and alters gut Microbiota in Apolipoprotein E-Deficient mice. J Am Heart Assoc. 2021;10(7):e019820. doi:10.1161/JAHA.120.019820. 33787322 PMC8174342

[21] Li W, Liu R, Li X, Tao B, Zhai N, Wang X, Li Q, Zhang Y, Gu W, Wang W, et al. Saxagliptin alters bile acid profiles and yields metabolic benefits in drug-naïve overweight or obese type 2 diabetes patient. J Diabetes. 2019;11(12):982–992. doi:10.1111/1753-0407.12956. 31141297

[22] Ma Q, Li Y, Wang M, Tang Z, Wang T, Liu C, Wang C, Zhao B. Progress in metabonomics of type 2 diabetes mellitus. Molecules. 2018;23(7):1834. doi:10.3390/molecules23071834. 30041493 PMC6100487

[23] Vettorazzi JF, Kurauti MA, Soares GM, Borck PC, Ferreira SM, Branco RCS, Michelone LSL, Boschero AC, Junior JMC, Carneiro EM. Bile acid TUDCA improves insulin clearance by increasing the expression of insulin-degrading enzyme in the liver of obese mice. Sci Rep. 2017;7(1):14876. doi:10.1038/s41598-017-13974-0. 29093479 PMC5665899

[24] Ito T, Yoshikawa N, Ito H, Schaffer SW. Impact of taurine depletion on glucose control and insulin secretion in mice. J Pharmacol Sci. 2015;129(1):59–64. doi:10.1016/j.jphs.2015.08.007. 26382103

[25] Aly HF, Mantawy MM. Comparative effects of zinc, selenium and vitamin E or their combination on carbohydrate metabolizing enzymes and oxidative stress in streptozotocin induced-diabetic rats. Eur Rev Med Pharmacol Sci. 2012;16(1):66–78. PMID: 22338550. 22338550

[26] Asmat U, Abad K, Ismail K. Diabetes mellitus and oxidative stress-A concise review. Saudi Pharm J. 2016;24(5):547–553. doi:10.1016/j.jsps.2015.03.013. 27752226 PMC5059829

[27] Feriani A, Tir M, Hachani R, Allagui MS, Tlili N, Nahdi S, Alwasel S, Harrath AH. Permethrin induced arterial retention of native and oxidized LDL in rats by promoting inflammation, oxidative stress and affecting LDL receptors, and collagen genes. Ecotoxicol Environ Saf. 2021;207:111269. doi:10.1016/j.ecoenv.2020.111269. 32911180

[28] de Boer Jf, Bloks VW, Verkade E, Heiner-Fokkema MR, Kuipers F, de Boer JF. New insights in the multiple roles of bile acids and their signaling pathways in metabolic control. Curr Opin Lipidol. 2018;29(3):194–202. doi:10.1097/MOL.0000000000000508. 29553998

[29] Agus A, Clément K, Sokol H. Gut microbiota-derived metabolites as central regulators in metabolic disorders. Gut. 2021;70(6):1174–1182. doi:10.1136/gutjnl-2020-323071. 33272977 PMC8108286

[30] Sun WL, Li XY, Dou HY, Wang XD, Li JD, Shen L, Ji HF. Myricetin supplementation decreases hepatic lipid synthesis and inflammation by modulating gut microbiota. Cell Rep. 2021;36(9):109641. doi:10.1016/j.celrep.2021.109641. 34469716

[31] Sharma M, Li Y, Stoll ML, Tollefsbol TO. The Epigenetic connection between the gut microbiome in obesity and diabetes. Front Genet. 2020;10:1329. doi:10.3389/fgene.2019.01329. 32010189 PMC6974692

[32] Sharma S, Tripathi P. Gut microbiome and type 2 diabetes: where we are and where to go? J Nutr Biochem. 2019;63:101–108. doi:10.1016/j.jnutbio.2018.10.003. 30366260

[33] Song Z, Cai Y, Lao X, Wang X, Lin X, Cui Y, Kalavagunta PK, Liao J, Jin L, Shang J, et al. Taxonomic profiling and populational patterns of bacterial bile salt hydrolase (BSH) genes based on worldwide human gut microbiome. Microbiome. 2019;7(1):9. doi:10.1186/s40168-019-0628-3. 30674356 PMC6345003

[34] Virtue AT, McCright SJ, Wright JM, Jimenez MT, Mowel WK, Kotzin JJ, Joannas L, Basavappa MG, Spencer SP, Clark ML, et al. The gut microbiota regulates white adipose tissue inflammation and obesity via a family of microRnas. Sci Transl Med. 2019;11(496):eaav1892. doi:10.1126/scitranslmed.aav1892. 31189717 PMC7050429

[35] Wu Q, Liang X, Wang K, Lin J, Wang X, Wang P, Zhang Y, Nie Q, Liu H, Zhang Z, et al. Intestinal hypoxia-inducible factor 2α regulates lactate levels to shape the gut microbiome and alter thermogenesis. Cell Metab. 2021;33(10):1988–2003.e7. doi:10.1016/j.cmet.2021.07.007. 34329568

[36] Hu M, Fok BS, Wo SK, Lee VH, Zuo Z, Tomlinson B. Effect of common polymorphisms of the farnesoid X receptor and bile acid transporters on the pharmacokinetics of ursodeoxycholic acid. Clin Exp Pharmacol Physiol. 2016;43(1):34–40. doi:10.1111/1440-1681.12490. 26382575

[37] Vujkovic-Cvijin I, Sklar J, Jiang L, Natarajan L, Knight R, Belkaid Y. (2020). Host variables confound gut microbiota studies of human disease. Nature, 587(7834), 448–454. 10.1038/s41586-020-2881-9 33149306 PMC7677204

[38] Nguyen T Loan, Vieira-Silva S, Liston A, Raes J. (2015). How informative is the mouse for human gut microbiota research?. Dis Model Mech, 8(1), 1–16. 10.1242/dmm.017400 25561744 PMC4283646

[39] Xiao JF, Zhou B, Ressom HW. Metabolite identification and quantitation in LC-MS/ms-based metabolomics. Trends Analyt Chem. 2012;32:1–14. doi:10.1016/j.trac.2011.08.009. PMC3278153 22345829

[40] Yang C, Du X, Hao R, Wang Q, Deng Y, Sun R. Effect of vitamin D3 on immunity and antioxidant capacity of pearl oyster Pinctada fucata martensii after transplantation: insights from LC-MS-based metabolomics analysis. Fish Shellfish Immunol. 2019;94:271–279. doi:10.1016/j.fsi.2019.09.017. 31499202

[41] Wiklund S, Johansson E, Sjöström L, Mellerowicz EJ, Edlund U, Shockcor JP, Gottfries J, Moritz T, Trygg J. Visualization of GC/TOF-MS-based metabolomics data for identification of biochemically interesting compounds using OPLS class models. Anal Chem. 2008;80(1):115–122. doi:10.1021/ac0713510. 18027910

[42] Simon MW, W Q, Jiang MM, Dalal EG, Bi SJ, Dong RJ. AnMBR as alternative to conventional CSTR to achieve efficient methane production from thermal hydrolyzed sludge at short HRTs. Energy. 2018;159:588–598. doi:10.1016/j.energy.2018.06.201.

[43] Magoč T, Salzberg SL. FLASH: fast length adjustment of short reads to improve genome assemblies. Bioinformatics. 2011;27(21):2957–2963. doi:10.1093/bioinformatics/btr507. 21903629 PMC3198573

[44] Caporaso JG, Kuczynski J, Stombaugh J, Bittinger K, Bushman FD, Costello EK, Fierer N, Peña AG, Goodrich JK, Gordon JI, et al. QIIME allows analysis of high-throughput community sequencing data. Nat Methods. 2010;7(5):335–336. doi:10.1038/nmeth.f.303. 20383131 PMC3156573

[45] Wang Q, Garrity GM, Tiedje JM, Cole JR. Naive Bayesian classifier for rapid assignment of rRNA sequences into the new bacterial taxonomy. Appl Environ Microbiol. 2007;73(16):5261–5267. doi:10.1128/AEM.00062-07. 17586664 PMC1950982

[46] Zhang Y, Gu Y, Ren H, Wang S, Zhong H, Zhao X, Ma J, Gu X, Xue Y, Huang S, et al. Gut microbiome-related effects of berberine and probiotics on type 2 diabetes (the PREMOTE study). Nat Commun. 2020;11(1):5015. doi:10.1038/s41467-020-18414-8. 33024120 PMC7538905

[47] Wang S, Ren H, Zhong H, Zhao X, Li C, Ma J, Gu X, Xue Y, Huang S, Yang J, et al. Combined berberine and probiotic treatment as an effective regimen for improving postprandial hyperlipidemia in type 2 diabetes patients: a double blinded placebo controlled randomized study. Gut Microbes. 2022;14(1):2003176. doi:10.1080/19490976.2021.2003176. 34923903 PMC8726654

