# Supplementary material for: Glycoursodeoxycholic acid regulates bile acids level and alters gut microbiota and glycolipid metabolism to attenuate diabetes
Source: Gut Microbes. 2023 Mar 26;15(1):2192155. doi: 10.1080/19490976.2023.2192155 (PMC10054359; doi:10.1080/19490976.2023.2192155)
Supplement: Supplemental Material [file KGMI_A_2192155_SM3043.zip › supplymentary material.docx]

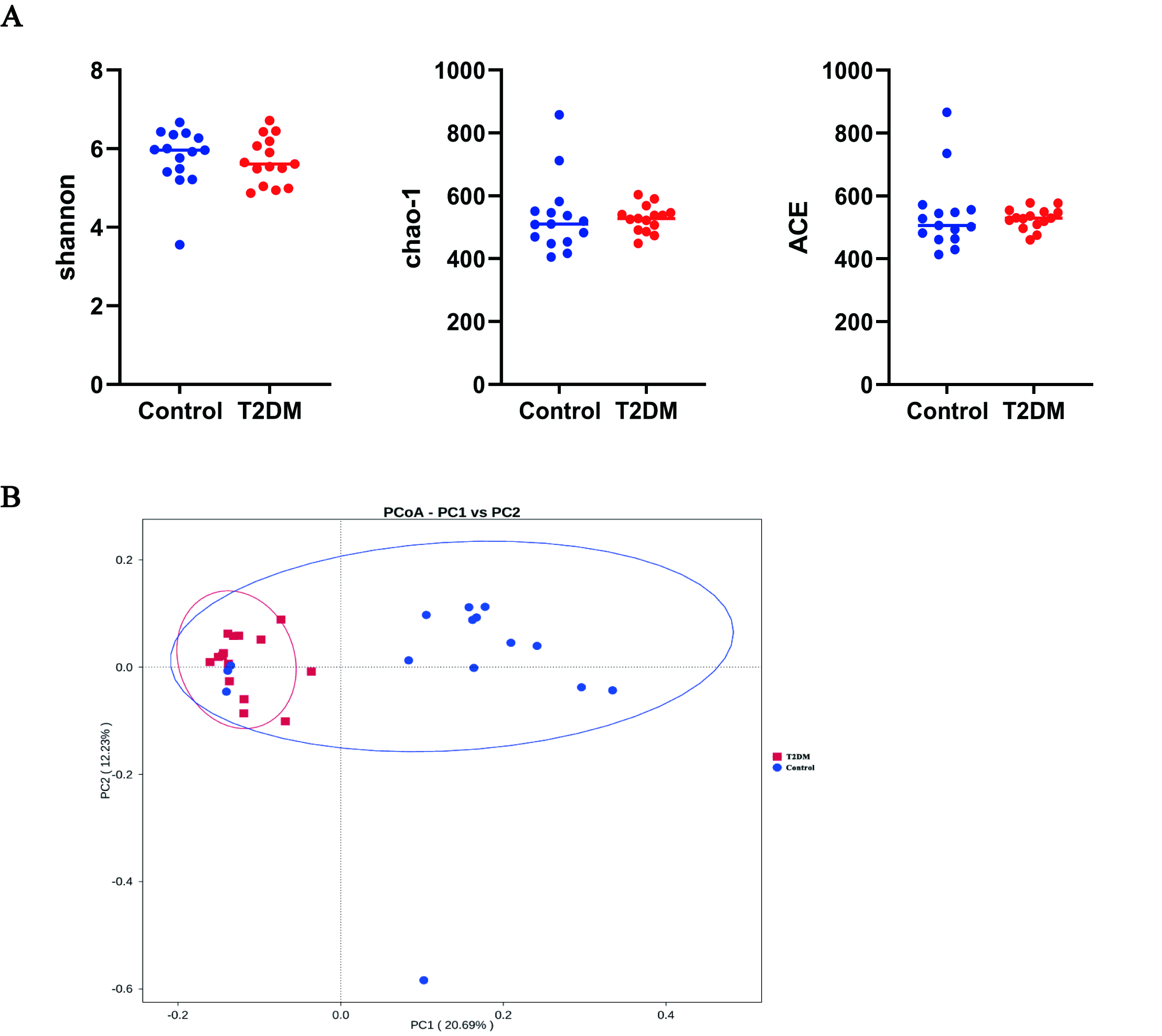


**Figure S1: α diversity and β diversity in T2DM and Control groups.** Related to Figure 1. A: α diversity: shannon, chao1 and ACE indices. B: Principle coordinate analysis (PCoA) plot generated using OTU metrics based on the Bray–Curtis similarity for control and T2DM groups. (n = 15 per group).


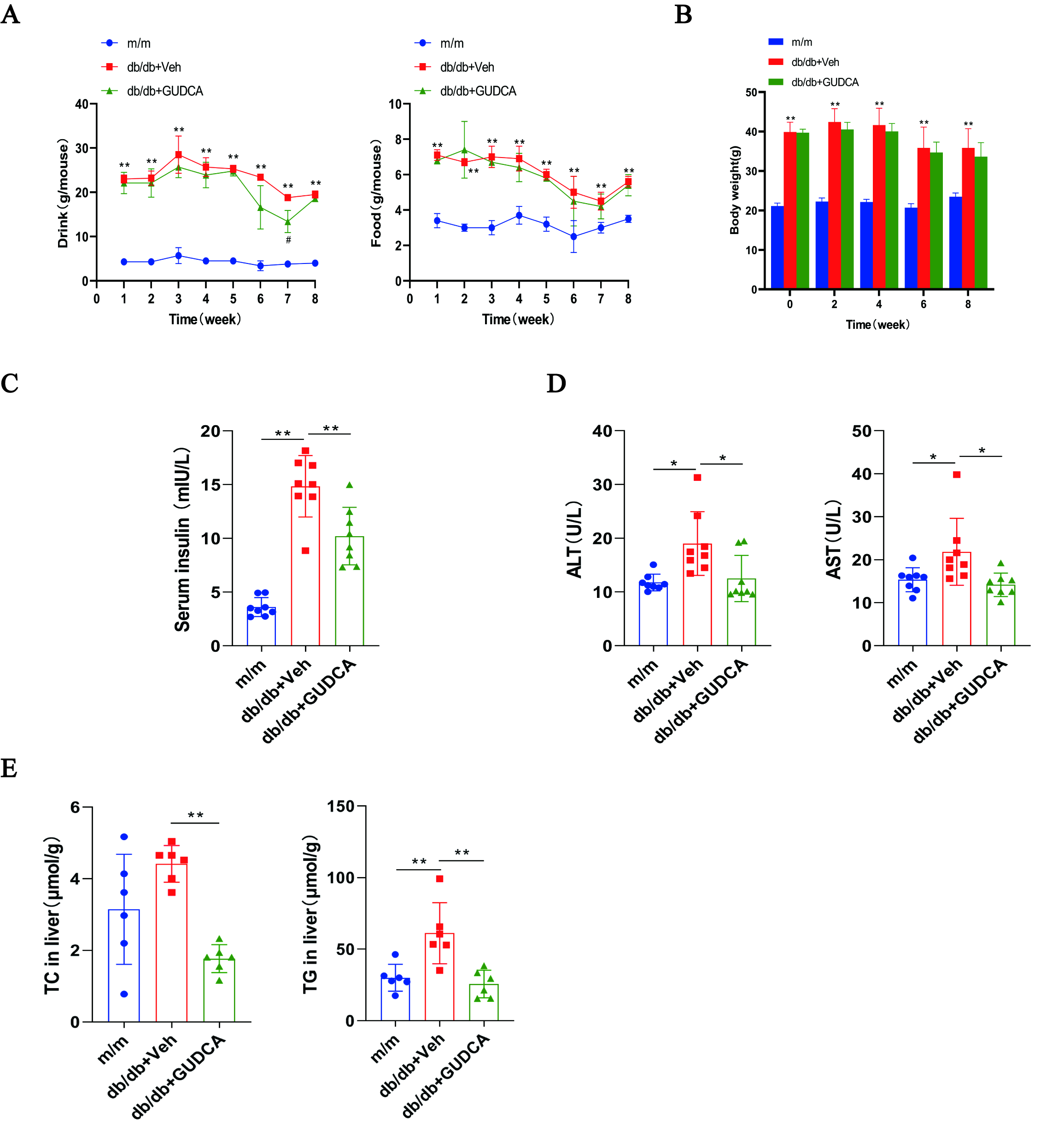


**Figure S2** **Physiological changes in m/m, db/db+Veh and db/db+GUDCA groups.** Related to Figure 2 and Figure 3. A: Drink and food intake. B: Body weight. n=10 mice in m/m group, n=9 mice in db/db+Veh and db/db+GUDCA groups. All P values were determined by two-tailed Student’s t-test, **p< 0.01 versus m/m; #p< 0.05 versus db/db+Veh. All data are presented as the mean ± SD.C: Serum insulin. D: ALT and AST. E: TC and TG in liver. n=8 mice/group. *p< 0.05, **p< 0.01. All data are presented as the mean ± SD.


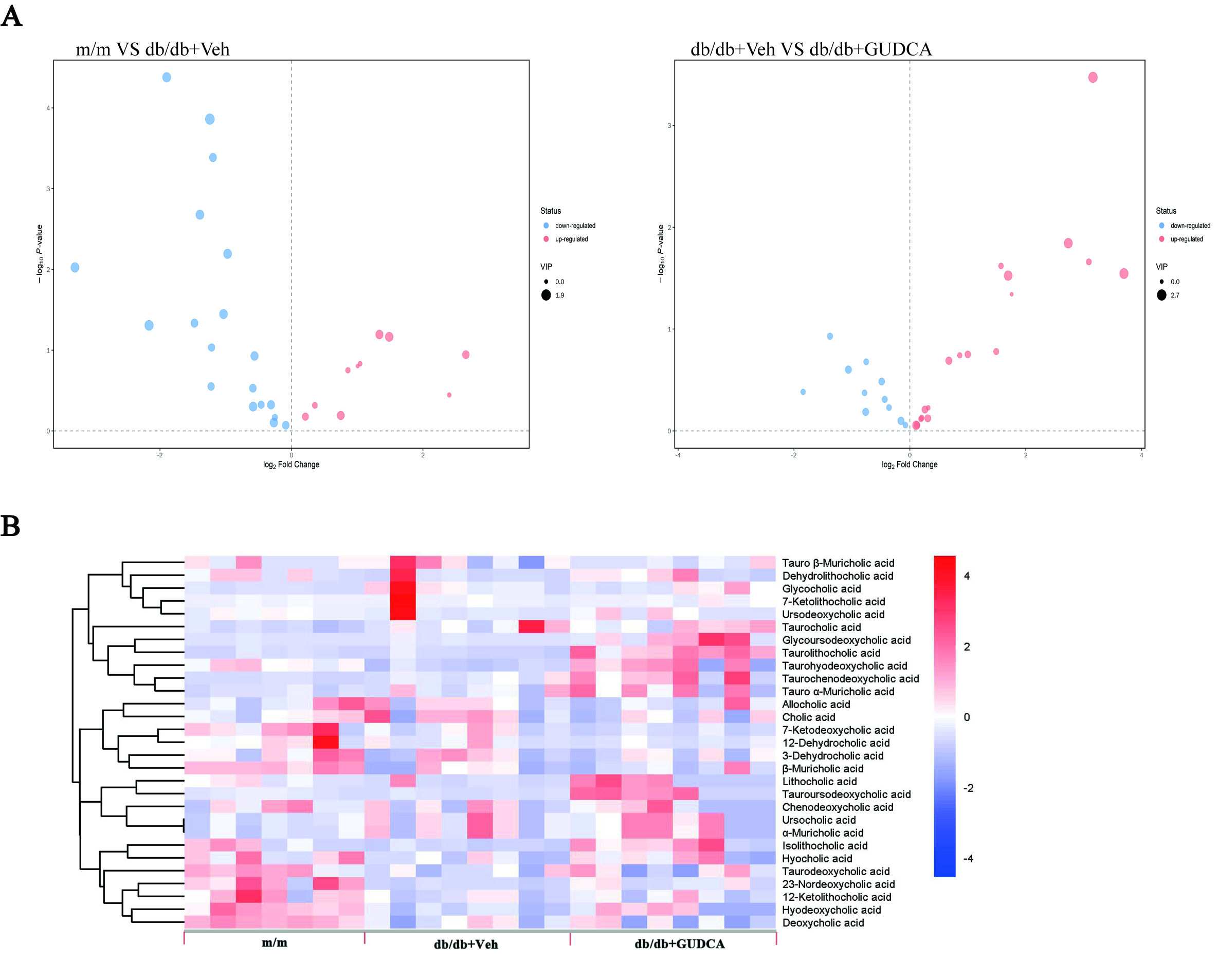


**Figure S3 Multivariate analysis metabolism in m/m, db/db+Veh and db/db+GUDCA groups.** Related to Figure 3. A: Volcano plots. Each point represents a metabolite, and the point size represents the VIP value of this metabolite in the OPLS–DA model. Red and blue indicate upregulation and downregulation, respectively. B: Hierarchical clustering analysis. The relative metabolite level is depicted according to the color scale. Red and blue indicate upregulation and downregulation, respectively.


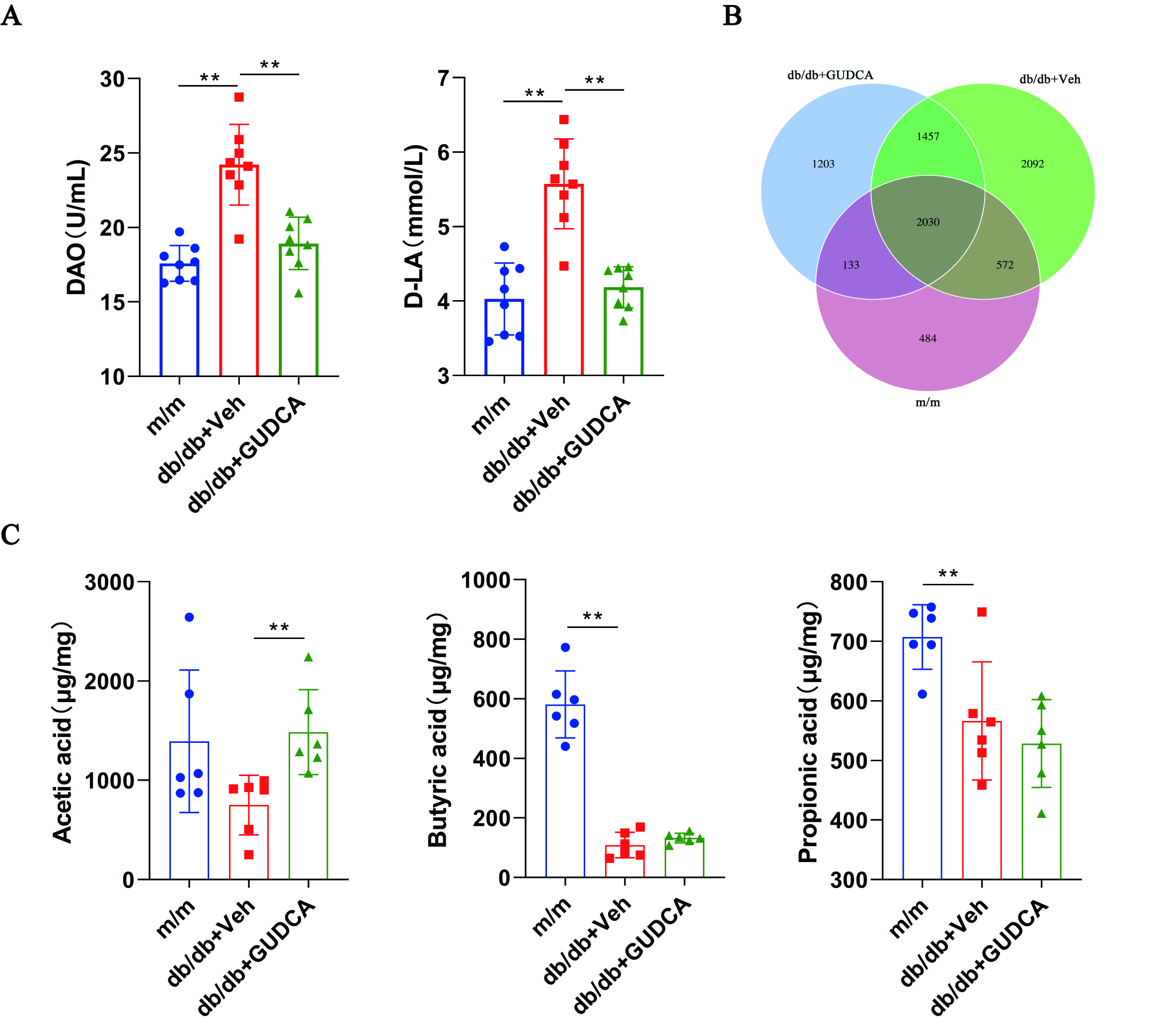


**Figure S4 Change in gut microbiota composition in m/m, db/db+Veh and db/db+GUDCA groups.** Related to Figure 4. A: DAO and D-LA in each group. B: Venn diagram, each circle in the graph represents a group. The numbers of the circles and the overlapping parts of the circles represent the number of OTUS shared by the groups. The numbers without overlapping parts represent the unique number of OTUS of the groups. C: Acetic acid, butyric acid and propionic acid in each group. All P values were determined by one-way ANOVA followed by the LSD test for multiple comparisons., *p< 0.05, **p< 0.01. All data are presented as the mean ± SD.

**Table S1** **Characteristics of the subjects with T2DM and control groups.** Related to Figure 1A. The values are presented as means ± standard deviation. Significant differences (*p<0.05, **p<0.01 and ***p<0.001) were analyzed using Two-tailed unpaired Student’s t-test.

|  | Control (n=50) | T2DM (n=30) |
| --- | --- | --- |
| Age (years) | 59.50±10.62 | 60.30±8.47 |
| BMI (kg/m^2^) | 25.74±4.42 | 26.78±4.32 |
| WHR | 0.91±0.09 | 0.93±0.11 |
| SBP (mmHg) | 123.26±23.01 | 142.73±19.73^*^ |
| DBP (mmHg) | 82.92±13.13 | 89.00±14.70 |
| TG (mmol/L) | 1.54±0.89 | 2.08±1.29^*^ |
| TC (mmol/L) | 5.09±1.00 | 5.72±1.12^*^ |
| LDL-C (mmol/L) | 2.62±0.90 | 3.10±0.85^*^ |
| HDL-C (mmol/L) | 1.47±0.32 | 1.36±0.21 |
| FBG (mmol/L) | 4.82±0.48 | 8.94±2.47^***^ |
| GLU (mmol/L) | 3.99±1.40 | 7.62±3.93^***^ |
| INS (mU/L) | 33.88±20.97 | 51.49±35.34^**^ |

**Table S2 Concentrations of serum bile acids in m/m, db/db+Veh and db/db+GUDCA groups. Related to Figure 4.** The values are presented as means ± standard error, n=7-8 for each group. Significant differences were analyzed using one-way ANOVA followed by the LSD test for multiple comparisons. *p< 0.05, versus m/m; #p< 0.05, ##p< 0.01, ###p<0.001 versus db/db+Veh.

| Bile acids | m/m | db/db+Veh | db/db+GUDCA |
| --- | --- | --- | --- |
| Dehydrolithocholic acid | 1.57±0.68 | 0.36±0.36 | 12.97±4.80(b) |
| Isoallolithocholic acid | - | - | 1.20±0.85 |
| Isolithocholic acid | 2.68±1.11 | - | 16.56±5.39^##^ |
| Lithocholic acid | 11.19±2.48 | 5.94±1.78 | 103.06±17.32^###^ |
| 23-Nordeoxycholic acid | 46.77±7.64 | 11.43±11.22^*^ | 20.01±4.00 |
| 7-Ketolithocholic acid | 0.63±0.44 | 2.49±1.60 | 7.62±4.74 |
| 12-Ketolithocholic acid | 23.24±4.55 | 21.45±10.09 | 26.12±5.58 |
| Apocholic acid | 0.44±0.44 | 3.43±2.72 | 2.33±1.18 |
| Ursodeoxycholic acid | 101.33±27.13 | 86.98±47.87 | 1332.94±525.34# |
| Hyodeoxycholic acid | 91.27±14.95 | 90.42±50.63 | 242.05±70.33 |
| Chenodeoxycholic acid | 25.07±9.73 | 63.06±35.74 | 158.85±64.81 |
| Deoxycholic acid | 806.00±122.75 | 819.35±260.93 | 1221.67±242.40 |
| Isodeoxycholic acid | 0.84±0.54 | - | 0.64±0.64 |
| 6,7-Diketolithocholic acid | 0.52±0.52 | 1.78±1.36 | 0.54±0.54 |
| 7-Ketodeoxycholic acid | 106.43±24.66 | 92.71±41.65 | 74.28±46.25 |
| 12-Dehydrocholic acid | 22.53±8.04 | 22.28±12.09 | 16.16±9.21 |
| 3-Dehydrocholic acid | 9.43±2.07 | 21.57±9.48 | 17.51±7.87 |
| Ursocholic acid | 28.44±5.90 | 200.87±109.28 | 242.31±82.90 |
| α-Muricholic acid | 28.33±6.06 | 205.49±112.18 | 244.41±84.05 |
| β-Muricholic acid | 713.00±80.34 | 1356.90±755.89 | 474.57±129.67 |
| Hyocholic acid | 3.37±0.65 | 3.75±1.55 | 11.61±3.84^#^ |
| Allocholic acid | 40.11±12.63 | 123.07±72.49 | 42.12±13.42 |
| Cholic acid | 787.49±157.44 | 2778.32±1297.89 | 1853.15±677.96 |
| Glycoursodeoxycholic acid | - | - | 27.68±13.45^#^ |
| Glycocholic acid | 2.43±0.64 | 19.86±6.27^*^ | 16.07±4.26 |
| Taurolithocholic acid | 0.28±0.28 | - | 10.03±1.56^###^ |
| Tauroursodeoxycholic acid | 63.02±12.48 | 55.78±16.02 | 1275.21±223.53^###^ |
| Taurohyodeoxycholic acid | 81.57±12.43 | 42.04±8.80 | 300.00±65.55^###^ |
| Taurochenodeoxycholic acid | 10.10±3.23 | 38.49±18.37 | 236.79±55.84^##^ |
| Taurodeoxycholic acid | 298.80±60.39 | 219.94±86.40 | 589.93±154.33^#^ |
| Tauro α-Muricholic acid | 72.27±19.28 | 181.70±87.54 | 844.24±279.86^#^ |
| Tauro β-Muricholic acid | 276.00±33.42 | 582.58±178.74 | 443.38±104.23 |
| Taurocholic acid | 468.25±96.24 | 1698.99±615.69 | 2303.64±570.29 |
