# Supplementary figures and images for: Glycoursodeoxycholic acid regulates bile acids level and alters gut microbiota and glycolipid metabolism to attenuate diabetes
Source: Gut Microbes. 2023 Mar 26;15(1):2192155. doi: 10.1080/19490976.2023.2192155 (PMC10054359; doi:10.1080/19490976.2023.2192155)

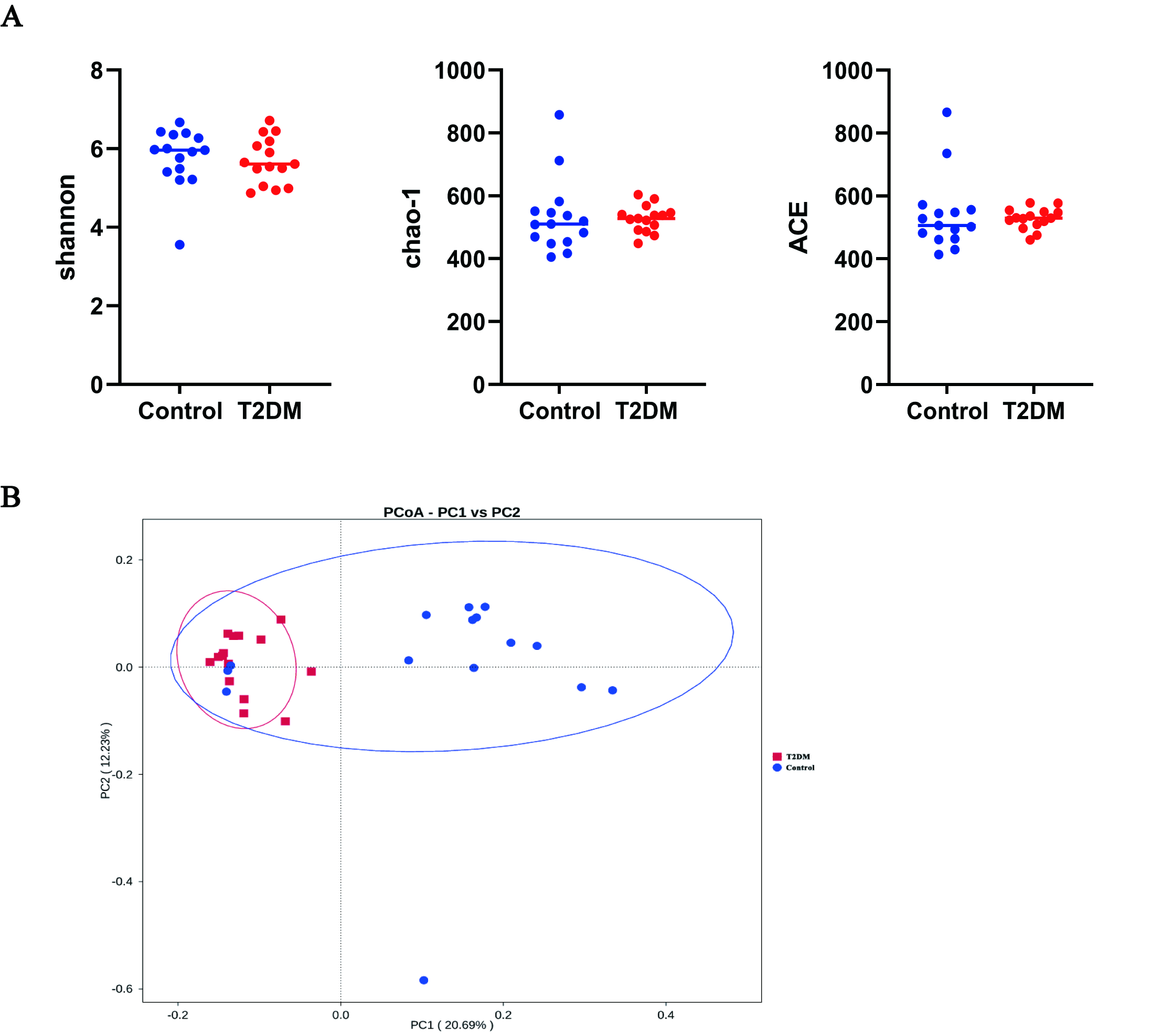

Supplement: Supplemental Material [file KGMI_A_2192155_SM3043.zip › FigureS1.tif]

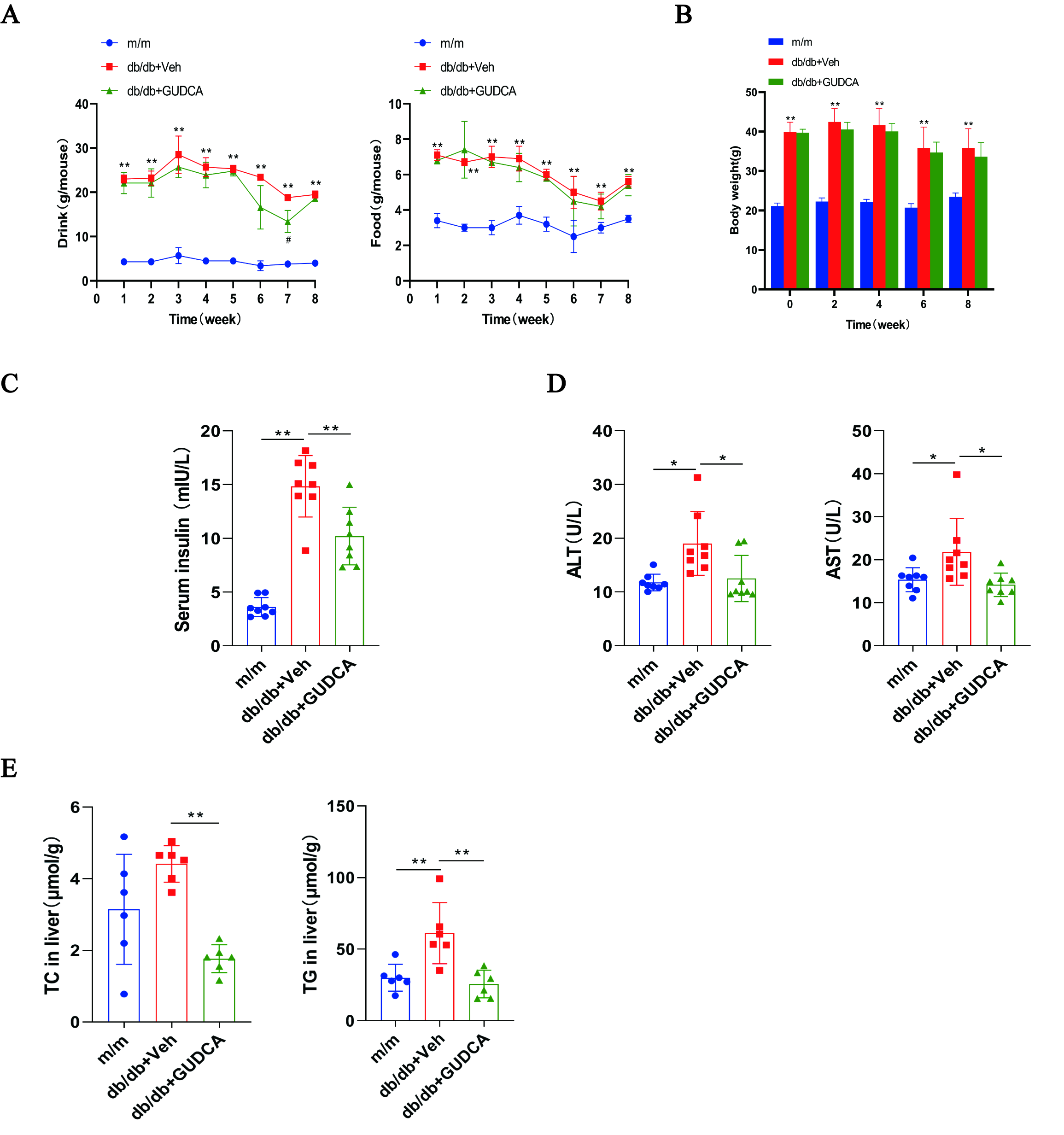

Supplement: Supplemental Material [file KGMI_A_2192155_SM3043.zip › FigureS2.tif]

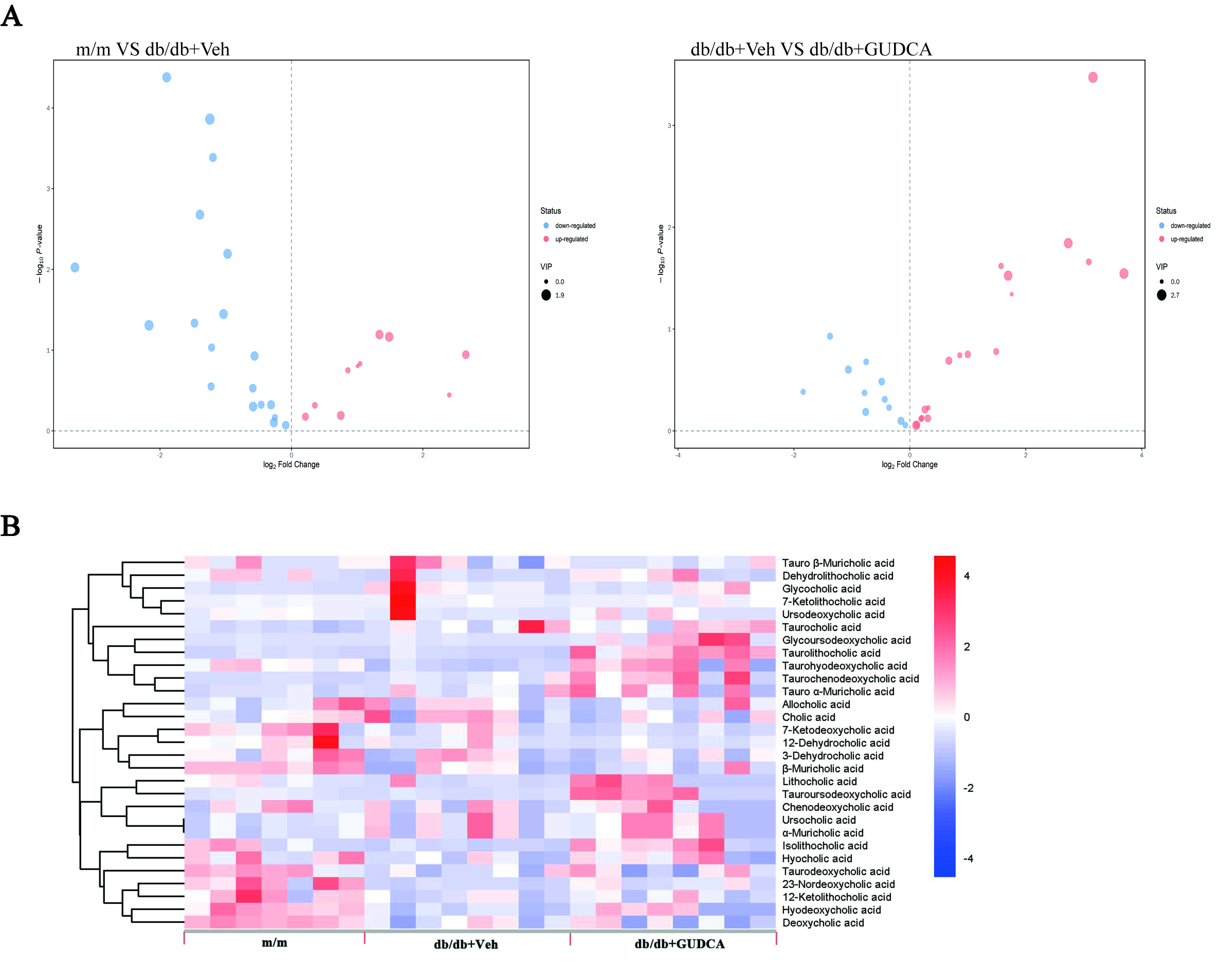

Supplement: Supplemental Material [file KGMI_A_2192155_SM3043.zip › FigureS3.tif]

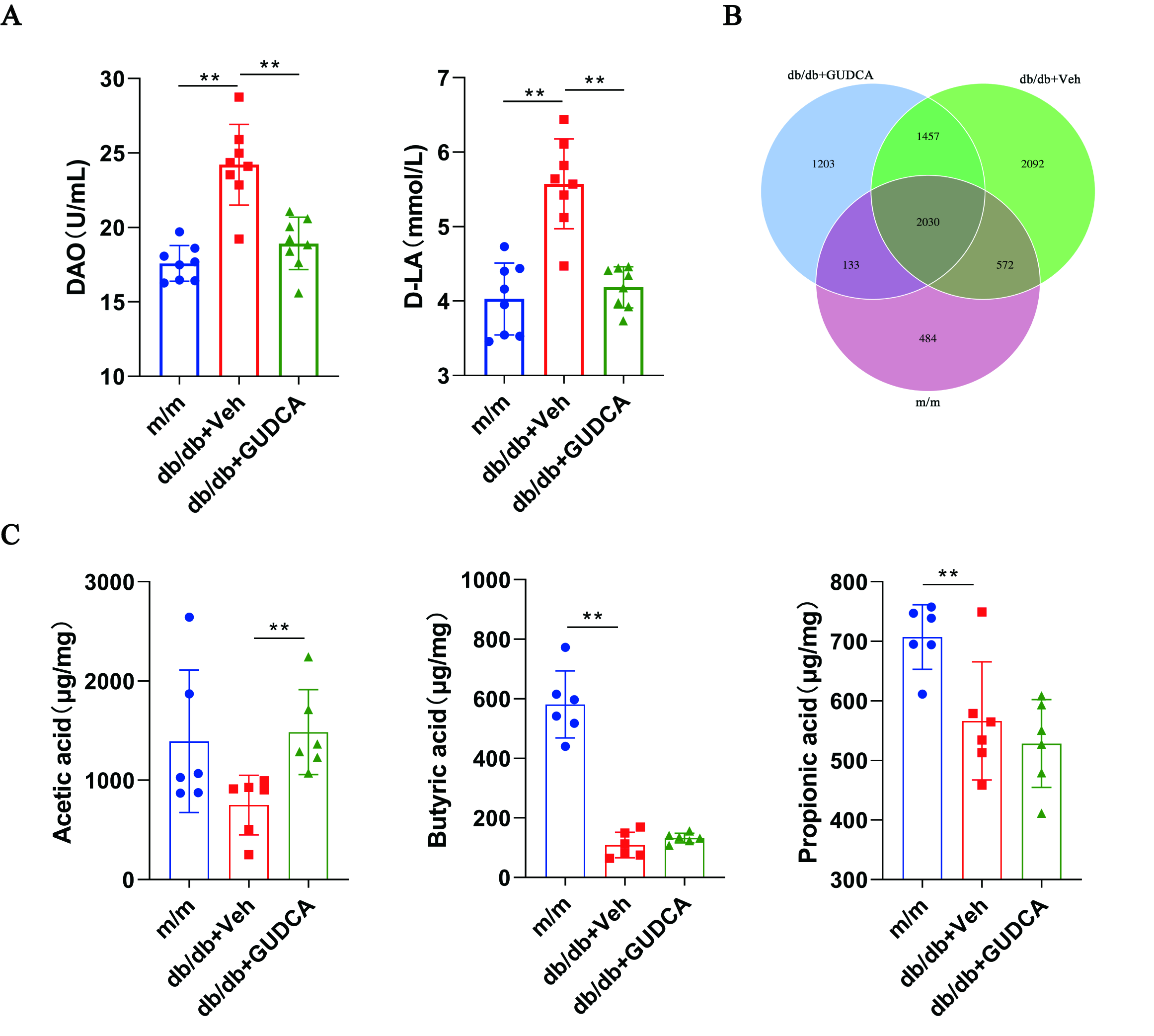

Supplement: Supplemental Material [file KGMI_A_2192155_SM3043.zip › FigureS4.tif]
